# Genetic diversity of *Mycobacterium tuberculosis* isolates from Beijing, China assessed by Spoligotyping, LSPs and VNTR profiles

**DOI:** 10.1186/1471-2334-12-372

**Published:** 2012-12-23

**Authors:** Bing Lu, Ping Zhao, Binbin Liu, Haiyan Dong, Qin Yu, Xiuqin Zhao, Kanglin Wan

**Affiliations:** 1 State Key Laboratory for Infectious Diseases Prevention and Control, National Institute for Communicable Disease Control and Prevention & National Tuberculosis Reference Laboratory, Chinese Center for Disease Control and Prevention, Beijing 102206, P. R. China; 2 Chaoyang Centre for Disease Control and Prevention, Beijing, 100021, P. R. China; 3 Hunan Provincial Institute for Tuberculosis Prevention and Control, Changsha, 410006, P. R. China; 4 University of South China, Hengyang, 421001, P. R. China

**Keywords:** *M. tuberculosis*, Beijing family, Genotyping

## Abstract

**Background:**

Tuberculosis is one of the most infectious diseases in the world. Molecular typing methods such as spoligotyping, and VNTR (variable number tandem repeats), IS*6110* in the NTF region and LSP (large sequence polymorphisms) analysis are generally useful tools for the resolution of various issues related to the classical epidemiology of *Mycobacterium tuberculosis* (*M. tuberculosis*).

**Methods:**

To determine the transmission characteristics of *M. tuberculosis* strains isolated in Beijing, China, and their genetic relationships, especially those among Beijing family strains, 260 *M. tuberculosis* strains isolated from patients presenting pulmonary tuberculosis were analyzed by spoligotyping, and by examining 22 VNTR loci and the presence/absence of IS*6110* in the NTF region, RD105 and RD181.

**Results:**

81% (211 strains) of the isolates studied were Beijing family strains, 174 (82.5%) of which were identified as modern Beijing strains based on the presence of IS*6110* upstream of the NTF region. RD181 was intact in 9 of the other 37 (17.5%) ancestral Beijing strains. The percentage of Beijing family strains in this study was consistent with previous reports. There are many differences, however, in allele diversity among VNTR loci between reports on strains from different areas.

**Conclusions:**

The Beijing family is the most prevalent genotype in Beijing city and the predominance of Beijing family strains has not altered in almost twenty years. Differences in the alleles and discrimination ability of VNTR loci between different regions is likely due to population differences in the regions where these *M. tuberculosis* strains were isolated or to differences in sampling times.

## Background

Tuberculosis (TB) remains one of the most serious infectious diseases in the world. The *Mycobacterium tuberculosis* (*M. tuberculosis*) lineage of greatest significance in China is the Beijing family. This lineage, which was first described in 1995 [1], has been reported to cause major outbreaks worldwide [2,3].

*M. tuberculosis* Beijing family strains can be recognized on the basis of highly conserved spoligotyping (spacer oligonucletide typing) patterns (spacers 1–34 are absent) [4-6], and characteristic IS*6110*-RFLP patterns containing a high number of bands [7]. Strains lacking one or more of the last nine spacers in the spoligotyping pattern are called Beijing-like strains [8].

Beijing family strains have been divided into two major groups, modern and ancient, based on a specific IS*6110* in the NTF region [6,9]. The group that has no IS*6110* in the NTF region is thought to be the most ancient [10].

As with other PCR-based genotyping techniques, VNTR (variable number tandem repeats) typing is a promising, quick and easy genotyping method and can be used for epidemiological studies of Beijing strains [11]. This method is based on size analysis of PCR-amplified VNTR loci, and it requires only basic PCR and agarose electrophoresis equipment [12]. The number of repeat copies per locus may vary among strains, and the use of several such loci has sufficient discriminating power [13]. VNTR profiles are presented as multiple digit numerical codes, each digit representing the copy number of a locus. However these VNTR loci present multiple independent genetic markers and are therefore ideally suited for evolutionary analyses [10].

LSPs (Large sequence polymorphisms), described in numerous studies of the *M. tuberculosis* complex, are reported to give important insights into its evolution and biology [2]. One LSP (RD105) has been observed in all Beijing family strains and ancestral spoligotyping pattern strains [14]. Additional LSPs (RD181) further divide the Beijing family into subgroups [15].

The aims of the study were to analyze the distribution of the tuberculosis lineage in Beijing city and to compare our results with those from other areas in China. In this study, we collected 260 *M. tuberculosis* clinical isolates from Beijing city in 2009, and genotyped them by spoligotyping, VNTR, IS*6110* in the NTF and LSP (RD105 and RD180) to provide some information of potential transmission and evolution.

## Methods

### *M. tuberculosis* clinical isolates

260 *M. tuberculosis* strains were collected from TB patients in Beijing, China in 2009. The patients were all diagnosed to have pulmonary tuberculosis according to the national guidelines of China, and were treated in local lung disease hospitals or tuberculosis hospitals. There was no direct transmission link between patients selected in this study. All patient samples were taken as part of standard care. Demographic data, including the identification number, sex, age, occupation, registered residence, current address, as well as results of mycobacterial smears, symptoms and treatment history were obtained from medical records provided by Chaoyang Centre for Disease Control and Prevention, Beijing.

### Ethics statement

The study was approved by the Ethics Committee of the National Institute for Communicable Disease Control and Prevention, Chinese Center for Disease Control and Prevention. All patients in the study signed informed consent forms.

### Culture of clinical isolates and DNA extraction

*M. tuberculosis* genomic DNA was extracted from mycobacterial colonies grown on Löwenstein-Jensen (LJ) medium by resuspending one loopful of mycobacterial colonies in 100 μl TE buffer (10 mM Tris-Cl, 1 mMEDTA) which was then incubated at 95°C for 15 min. Supernatants containing the DNA were collected by centrifugation at 12,000 g for 3 min and stored at −20°C for further use.

### Genotyping methods

#### Spoligotyping

Spoligotyping which relies on the highly polymorphic DR (direct repeat) locus in the genome of *M. tuberculosis,* is a novel method for defining the Beijing family. Spoligotyping was performed according to the method described by Kamerbeek et al. [5].

#### VNTR

22 VNTR loci, including 5 exact tandem repeat (ETR A,B,C,D and E) loci, 7 mycobacterial interspersed repetitive-units (MIRU10,16,23,26,27,39 and 40), 4 Mtub loci (Mtub4, 21, 30 and 39), 5 Qub (Queens’s University of Belfast) loci (Qub11a, 11b, 18, 26, and 4156), and VNTR3820 locus, were analyzed in this study. PCR products were analyzed on 12 cm 2% agarose gels with 11 lanes. Each gel included two 100-bp DNA Markers (one on either side of the gel), an H37Rv sample as a positive control, and 8 samples. The copy number at each locus was calculated by BioNumerics software. Determination of the discriminatory power of the VNTR loci was calculated using the Hunter-Gaston discriminatory Index (HGDI) [16]. The HGDI was calculated using the following formula: $\text{HGDI}=1\left[\frac{1}{N\left(N-1\right)}\sum_{j=1}^{\text{s}}\mathrm{nj}\left(\mathrm{nj}-1\right)\right]$, where HGDI is the numerical index of discrimination, N is the total number of strains in the typing scheme, s is the total number of different strain types, and nj is the number of strains belonging to the jth type [17,18].

#### IS*6110* in the NTF region

The IS*6110* in the NTF region was analyzed according to methods used in previous studies [19]. All Beijing family strains identified by the spoligotyping method were amplified by PCR to detect the presence or absence of IS*6110* in the NTF region. PCR analysis was performed using the following oligonucleotide primers: MDR-6, 5′-CCAGATATCGGGTGTGTCGAC-3′; MDR-6r, 5′-TGCCGTTGTCGAAATCTAAACCC-3′. Strains with the insert yielded an amplified product of ≈1,800-bp, while those without the insert yielded an ≈700-bp PCR product [19,20].

#### Large sequence polymorphisms (LSPs)

RD105, and RD181 were analyzed in all the Beijing family strains from our study population, using a method described previously [15]. PCR analysis was performed using the following oligonucleotide primers: RD105L, 5′-GGAGTCGTTGAGGGTGTTCATCAGCTCAGTC-3′; RD105R, 5′-CGCCAAGGCCGCATAGTCACGGTCG-3′; RD181L, 5′-CGCAACGGCCGCGGTGAACTCT-3′; RD181R, 5′-CGGGCGGCTGCGGGAACCTT-3′.

### Data analysis

Spoligotypes in binary format were entered in an Excel spreadsheet and compared with the spoligotyping database SpolDB4 (http://www.pasteur-guadeloupe.fr:8081/SITVITDemo/index.jsp) [21]. BioNumerics software version 5.0 (Applied Maths, Belgium) was used for data analysis and generating a cluster map. Clustering was based on VNTR results, and was performed using the categorical co-efficient and UPGMA in BioNumerics 5.0.

## Results

### Spoligotyping

Of the 260 isolates, 246 (94.62%) were classified into one of the 17 shared international types (SITs) using SpolDB4.0 (Table 1 and Additional file 1: Table S1). The remaining 14 isolates generated 12 different spoligotypes that have not previously been described in the database. The most frequent strains were SIT1 (77.3%), followed by SIT53 (6.92%) and SIT190 (1.92%). 211 strains (81%) belonged to either the Beijing family (including SIT1, SIT190, SIT1364 and two new types), or the Beijing-like family (including SIT269, SIT632, and SIT255).

**Table 1:** **Spoligotype shared types for the 260** ***M. tuberculosis *strains evaluated in this study.**

**SIT n**^**a**^	**Spoligotype description binary**	**SpolDB4 ID**^**b**^	**No.**^**c**^	**Prevalence (%)**^**d**^
1	□□□□□□□□□□□□□□□□□□□□□□□□□□□□□□□□□□ ■ ■ ■ ■ ■ ■ ■ ■ ■	Beijing	197	77.3%
53	■ ■ ■ ■ ■ ■ ■ ■ ■ ■ ■ ■ ■ ■ ■ ■ ■ ■ ■ ■ ■ ■ ■ ■ ■ ■ ■ ■ ■ ■ ■ ■□□□□ ■ ■ ■ ■ ■ ■ ■	T1	18	6.92%
190	□□□□□□□□□□□□□□□□□□□□□□□□□□□□□□□□□□ ■ ■ ■ ■ ■□ ■ ■ ■	Beijing	5	1.92%
52	■ ■ ■ ■ ■ ■ ■ ■ ■ ■ ■ ■ ■ ■ ■ ■ ■ ■ ■ ■ ■ ■ ■ ■ ■ ■ ■ ■ ■ ■ ■ ■□□□□ ■ ■ ■□ ■ ■ ■	T2	5	1.92%
	■ ■ ■ ■ ■ ■ ■ ■ ■ ■ ■ ■ ■□□□□□□□□□□□ ■ ■ ■ ■ ■ ■ ■ ■□□□□ ■ ■ ■ ■ ■ ■ ■	New	4	0.3%
154	■ ■ ■ ■□ ■ ■ ■ ■ ■ ■ ■ ■ ■ ■ ■ ■ ■ ■ ■ ■ ■ ■ ■ ■ ■ ■ ■ ■ ■ ■ ■□□□□ ■ ■ ■ ■ ■ ■ ■	T1	3	1.15%
1364	□□□□□□□□□□□□□□□□□□□□□□□□□□□□□□□□□□ ■ ■ ■□□ ■ ■ ■ ■	Beijing	2	0.77%
269	□□□□□□□□□□□□□□□□□□□□□□□□□□□□□□□□□□□□ ■ ■ ■ ■ ■ ■ ■	Beijing-like	2	0.77%
917	■ ■ ■ ■ ■ ■ ■ ■ ■ ■□ ■ ■ ■ ■ ■ ■ ■ ■ ■ ■ ■ ■ ■ ■ ■ ■ ■ ■ ■ ■ ■□□□□ ■ ■ ■ ■ ■ ■ ■	T1	2	0.77%
1688	■ ■ ■ ■ ■ ■ ■ ■ ■ ■ ■ ■ ■ ■ ■ ■ ■ ■ ■□□□□□□ ■ ■ ■ ■ ■ ■ ■□□□□ ■ ■ ■ ■ ■ ■ ■	T1	2	0.6%
73	■ ■ ■ ■ ■ ■ ■ ■ ■ ■ ■ ■□ ■ ■ ■ ■ ■ ■ ■ ■ ■ ■ ■ ■ ■ ■ ■ ■ ■ ■ ■□□□□ ■ ■ ■□ ■ ■ ■	T2-T3	2	0.77%
632	□□□□□□□□□□□□□□□□□□□□□□□□□□□□□□□□□□ ■ ■ ■□ ■ ■ ■ ■ ■	Beijing	1	0.38%
	□□□□□□□□□□□□□□□□□□□□□□□□□□□□□□□□□□ ■ ■ ■□□□ ■ ■ ■	New(Beijing?)	1	0.38%
255	□□□□□□□□□□□□□□□□□□□□□□□□□□□□□□□□□□ ■ ■ ■ ■□ ■ ■ ■ ■	Beijing	1	0.38%
	□□□□□□□□□□□□□□□□□□□□□□□□□□□□□□□□□□ ■ ■□□□□□□□	New(Beijing?)	1	0.38%
1311	□□□□□□□□□□□□□□□□□□□□□□□□□□□□□□□□□□ ■ ■ ■ ■ ■□□□□	U	1	0.38%
	■□ ■ ■ ■ ■ ■ ■ ■ ■ ■ ■ ■ ■ ■ ■ ■ ■ ■ ■ ■ ■ ■ ■ ■ ■ ■ ■ ■ ■□□□□□□ ■ ■ ■ ■ ■ ■ ■	New	1	0.38%
	■□ ■ ■ ■ ■ ■ ■ ■ ■ ■ ■ ■ ■ ■ ■ ■ ■ ■ ■ ■ ■ ■ ■□ ■ ■ ■ ■ ■□□□□□□ ■ ■ ■□ ■ ■ ■	New	1	0.38%
1163	■ ■□ ■ ■ ■ ■ ■ ■ ■ ■ ■□ ■ ■ ■ ■ ■ ■ ■ ■ ■ ■ ■ ■ ■ ■ ■ ■ ■ ■ ■□□□□ ■ ■ ■ ■ ■ ■ ■	T3	1	0.38%
	■ ■ ■□□□□ ■ ■ ■ ■ ■ ■ ■ ■ ■ ■ ■ ■ ■ ■ ■□□□□□□□□□□□□□□ ■ ■ ■ ■ ■ ■ ■	New	1	0.38% 5
	■ ■ ■ ■ ■□□ ■ ■ ■ ■ ■ ■ ■ ■ ■ ■ ■ ■ ■ ■ ■ ■ ■ ■ ■ ■ ■ ■ ■□□□□□□ ■ ■ ■□ ■ ■ ■	New	1	0.38%
	■ ■ ■ ■ ■□ ■ ■ ■ ■ ■□□□□□□□□□□□□□□□□□□□□□□□□□ ■ ■ ■ ■ ■ ■ ■	New	1	0.38%
500	■ ■ ■ ■ ■ ■ ■ ■ ■ ■ ■ ■□□□□□□□□□□□□□□ ■ ■ ■ ■ ■ ■□□□□ ■ ■ ■ ■ ■ ■ ■	T1	1	0.38%
	■ ■ ■ ■ ■ ■ ■ ■ ■ ■ ■ ■□□□□□□□□□ ■ ■ ■ ■ ■ ■ ■ ■ ■ ■ ■□□□□ ■ ■ ■ ■ ■ ■ ■	Nwe	1	0.38%
	■ ■ ■ ■ ■ ■ ■ ■ ■ ■ ■ ■ ■□□□□□□□□□□□□□□□□□□□□□□□□□□□□□□	U	1	0.38%
1580	■ ■ ■ ■ ■ ■ ■ ■ ■ ■ ■ ■ ■ ■ ■ ■ ■ ■ ■ ■ ■ ■□□ ■ ■ ■ ■ ■ ■ ■ ■□□□□ ■ ■ ■ ■ ■ ■ ■	T1	1	0.38%
	■ ■ ■ ■ ■ ■ ■ ■ ■ ■ ■ ■ ■ ■ ■ ■ ■ ■ ■ ■ ■ ■ ■□□□□ ■ ■ ■ ■ ■□□□□ ■ ■ ■□ ■ ■ ■	New	1	0.38%
	■ ■ ■ ■ ■ ■ ■ ■ ■ ■ ■ ■ ■ ■ ■ ■ ■ ■ ■ ■ ■ ■ ■□ ■□□ ■ ■ ■ ■ ■□□□□ ■ ■ ■ ■ ■ ■ ■	New	1	0.38%
54	■ ■ ■ ■ ■ ■ ■ ■ ■ ■ ■ ■ ■ ■ ■ ■ ■ ■ ■ ■ ■ ■ ■ ■ ■ ■ ■ ■ ■ ■ ■ ■□□ ■ ■ ■ ■ ■ ■ ■ ■ ■	MANU2	1	0.38%

**a **SIT from SpolDB4.0.

**b **Representing spoligotype families as assigned in SpolDB4.0.

**c **Number of isolates with a common SIT.

**d **Prevalence represents the number of isolates with a common SIT relative to the total number of isolates in this study.

### Allelic diversity of the VNTR loci

**Figure 1:**
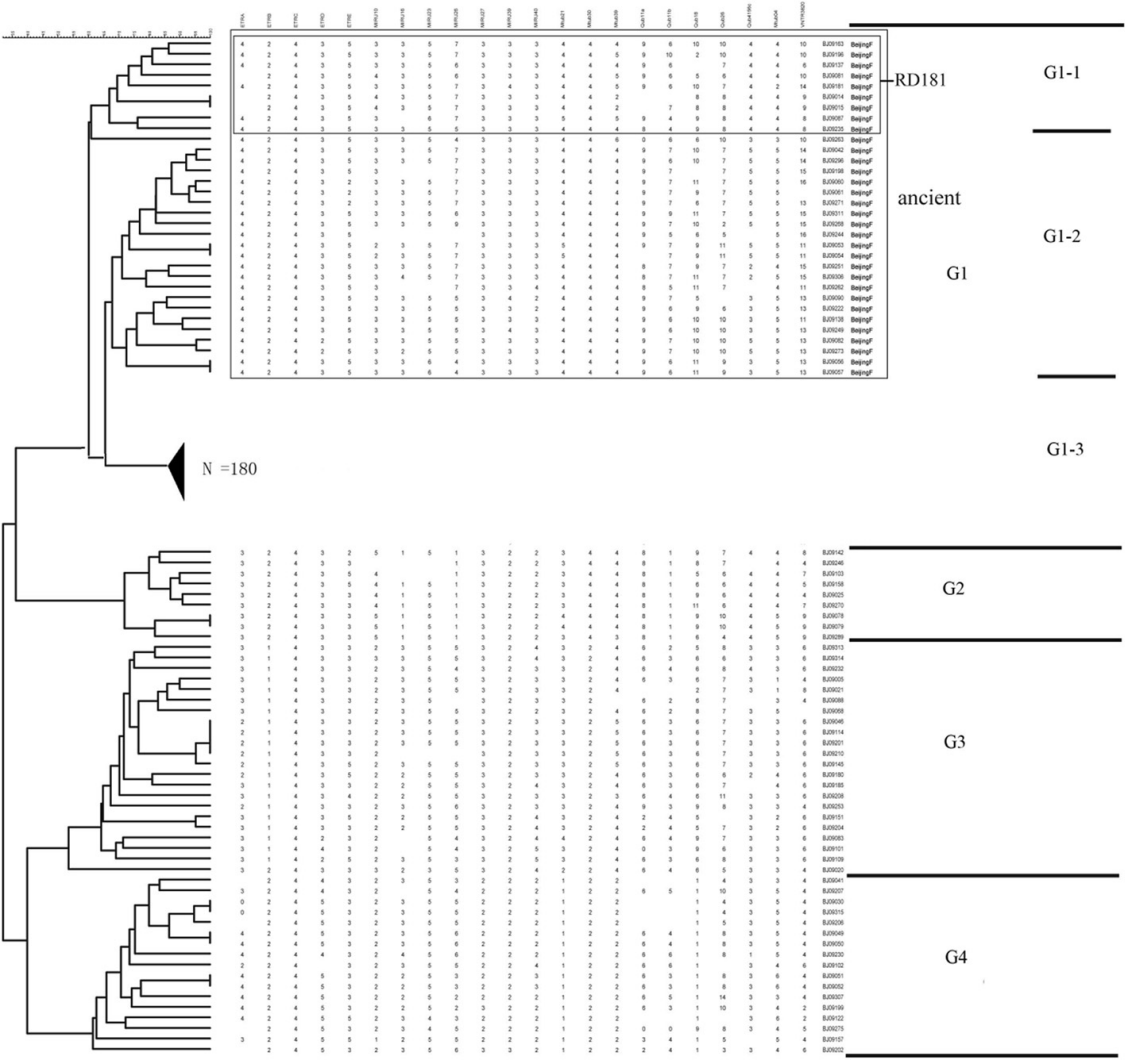
**Cluster map based on 22 VNTR loci in 260** ***M. tuberculosis *strains, clustered using the UPGMA (unweighted pair group cluster method with arithmetic mean) method in BioNumerics 5.0.** The large box shows ancient Beijing strains, while the small box shows the 9 strains in which RD181 is intact.

We analyzed all 260 isolates by screening 22 VNTR loci to investigate the genotypic diversity of the population in detail. This genotyping method is used as a standard discrimination tool for *M. tuberculosis* because of its high powers of resolution among isolates from cosmopolitan origins [13]. VNTR results are shown in Additional file 1: Table S1 and VNTR profiles analyzed in this study are listed in Table 2. The cluster map in Figure 1 of the 260 *M. tuberculosis* strains was generated by BioNumerics 5.0. The 260 strains were classified as G1 to G4 strains using the cluster cutoff value calculated by the BioNumerics software according to VNTR results. G1 strains were further divided into three subgroups, G1-1, G1-2, and G1-3 (Figure 1). VNTR loci differed significantly in their allelic diversity and power of discrimination (Table 2). The VNTR3820 locus showed the highest allelic diversity among the 260 strains (0.851), while the ETRC locus showed the lowest allelic diversity (0.03).

**Table 2:** **Diversity of VNTR loci and their profiles in all *M. tuberculosis *strains and Beijing family strains from Beijing city, Jiangsu and Shanghai (references).**

**Locus**	**Hunter-Gaston Index (total strains) N=260**	**No. of alleles (range)**	**Hunter-Gaston Index Beijing Family N=211**	**No. of alleles (Beijing family) (range)**	**Locus diversity of Beijing family strains in area of isolation**		
					**Beijing (Previous) **[24]** N=72 [alleles]**	**Jiangsu **[23]** N=209**	**Shanghai **[22]** (Chongming) N=65**
**VNTR3820**	0.851	19 (2–22)	0.789	15 (6–22)			0.85
**Qub11b**	0.747	10 (1–10)	0.646	9 (1–10)	0.651 [5(3–7)]	0.625	0.65
**Qub18**	0.696	10 (1–11)	0.613	10 (1–11)			0.65
**Qub11a**	0.675	9 (2–11)	0.598	8 (3–11)			0.61
**Qub26**	0.654	12 (1–14)	0.57	11 (1–11)	0.518 [8(3–10)]	0.613	0.60
**Mtub21**	0.58	7 (1–7)	0.393	6 (1–7)	0.556 [5(1–6)]	0.535	0.52
**MIRU26**	0.568	9 (1–9)	**0.373**	7 (3–9)	0.353 [6(3–9)]	0.560	**0.61**
**Qub4156c**	0.343	5 (1–5)	0.297	4 (2–5)	0.395 [5(1–5)]	0.203	0.49
**MIRU16**	0.381	4 (1–4)	**0.265**	3 (2–4)	**0.068 [3(2–4)]**	0.262	0.24
**Mtub04**	0.435	6 (1–6)	0.265	5 (2–6)	0.306 [4(2–5)]	0.426	0.30
**ETRE**	0.415	5 (2–6)	**0.229**	5 (2–6)	0.169 [3(4–6)]	**0.668**	0.25
**ETRA**	0.418	3 (2–4)	0.224	2 (3–4)	0.232 [3(2–4)]	0.201	0.03
**MIRU10**	0.434	5 (1–5)	0.21	4 (1–4)	0.144 [3(1–3)]	0.262	0.20
**Mtub39**	0.332	6 (2–7)	0.204	6 (2–7)	0.171 [5(1–6)]	0.213	0.06
**MIRU23**	0.194	5 (2–6)	**0.179**	**5 (2–6)**	**0.014 [2(5–6)]**	0.250	0.06
**MIRU40**	0.356	5 (1–5)	0.171	5 (1–5)	0.194 [4(1–4)]	0.276	0.15
**ETRD**	0.259	5 (1–5)	0.152	4 (1–4)	0.120 [4(0–3)]	0.536	0.06
**MIRU39**	0.374	4 (1–4)	0.127	4 (1–4)	0.119 [3(2–4)]	0.178	0.29
**Mtub30**	0.304	3 (2–4)	0.065	3 (2–4)	0.068 [4(2–5)]	0.196	0.09
**MIRU27**	0.15	4 (1–4)	0.056	4 (1–4)		0.084	0.03
**ETRB**	0.182	3 (1–3)	0.038	2 (1–2)	0.014 [2(1–2)]	0.056	0
**ETRC**	0.03	3 (2–4)	0.028	3 (2–4)	0.094 [4(2–6)]	0.066	

The VNTR profiles of Beijing family strains analyzed in this and previous studies were combined and are listed in Table 2. The VNTR3820 locus showed the highest allelic diversity among the 211 Beijing family strains (0.789), while the ETRC locus showed the lowest allelic diversity (0.028). Comparing the allelic diversity observed here with that of previous studies carried out in regions such as Jiangsu province or Shanghai city, both of which are located in the south of China [22,23], we found more differences in the allelic diversity of some loci (for example, ETRE and MIRU26). Furthermore, in a previous study on Beijing city, published by Jiao et al. four years ago [24], the alleles and discrimination power of MIRU16 and MIRU23 were very different to that observed in this study (Table 2). For instance, we obtained an HGDI for MIRU16 of 0.265, while Jiao et al., obtained a HGDI of 0.068; we observed five MIRU23 alleles (2, 3, 4, 5, 6), while only 2 alleles (5, 6) were observed by Jiao et al.

### NTF and LSP

The RD105 deletion was found in 211 Beijing family strains. Of the 211 epidemiologically unlinked Beijing MTB isolates, 174 (82.5%) were identified as modern Beijing strains based on the presence of IS*6110* upstream of the NTF region. In the remaining 37 (17.5%) ancestral Beijing strains, 9 strains had an intact RD181.

The definition of the phylogenetic sublineages of the 260 *M. tuberculosis* isolates from Beijing is listed in Table 3. G1 strains are Beijing family strains and have an RD105 deletion. G1-1 subgroup strains have an intact RD 181. The G1-1 and G1-2 subgroups are ancient Beijing family strains (Table 3).

**Table 3:** **Definition of the phylogenetic sublineages of the 260** ***M. tuberculosis *isolates from Beijing.**

**Sublineage**		**Spoligotyping**	**LSP**		**IS*6110 *in NTF**
			**RD105**	**RD181**	
G1	G1-1	Beijing family	Deletion	Intact	Ancient
	G1-2	Beijing family	Deletion	Deletion	Ancient
	G1-3	Beijing family	Deletion	Deletion	Modern
G2		Non-Beijing	Intact	---	---
G3		Non-Beijing	Intact	---	---
G4		Non-Beijing	Intact	---	---

* In RD105 and RD181 columns, ‘1’ means the RD is intact; ‘0’ means the RD is deleted.

## Discussion

Molecular typing methods are generally useful tools for resolving various issues related to the classical epidemiology of human pathogens, including *M. tuberculosis*. Spoligotyping is definitely a “gold standard” method for detecting a Beijing family strain of *M. tuberculosis*[10]. The Beijing genotype was identified for the first time in strains isolated in the Beijing area of China in 1995, giving rise to the name of the genotype [1].

Beijing family strains having a characteristic spoligotype pattern (loci 1–34 are absent; loci 35–43 are present) have been identified in many areas. Moreover, in an *M. tuberculosis* molecular epidemiological investigation in China, Beijing family strains were the most prevalent lineage in China (74.08%), based on the SpolDB4.0 spoligotype database and SpotClust results [25]. In this study 211 strains were Beijing family strains, accounting for 81% of all isolates examined.

Interestingly, in the first molecular epidemiology study in Beijing city, 89.4% of the *M. tuberculosis* strains collected from 1992 to 1994 were identified as Beijing family strains [1], and 85.1% of strains collected in Beijing from 2002 to 2005 were also identified as Beijing family strains [24]. Is this study of isolates collected in 2009, 81% of the strains were Beijing family strains. Since there is no statistically significant difference between these three percentages, as determined using the *X*^2^ test (p > 0.05) we conclude that the predominance of Beijing family strains has not altered for almost twenty years.

There is some debate over the use of RD181 for classifying isolates. An early study showed that RD181 can be used to divide Beijing isolates into ancestral (RD181 intact) and modern (RD181 deleted) strains [26]. Faksri et al., however, suggested that RD181 alone does not provide sufficient discrimination to define ancestral or modern Beijing lineages, but with RD181 bing intact or deleted ancestral Beijing family strains can be divided into two subgroups [27]. Results from this study are consistent with this report in that ancestral Beijing family strains could be divided into RD181 intact strains (9 strains) and RD181 deletion strains (28 strains).

During the process of evolution in Beijing family strains, numbers of VNTR repeats can increase or decrease. Changes in the numbers of repeats in VNTRs can differ with locus. It has been shown that expansion and contraction in the number of repeats of VNTRs can occur during the evolutionary process [28]. Many previous studies have shown that VNTR loci can be good markers for phylogenetic estimation [29]. In this study, we found that the number of Mtub21 repeats was different between ancient and modern Beijing family strains, with the number of repeats being much lower in non-Beijing family strains. In agreement with other reports, we conclude that Mtub21 is an evolutionarily informative VNTR locus [30].

Many previous studies have suggested that VNTR loci vary in their ability to discriminate Beijing genotype strains from geographically distant areas [11,22-24] (Table 2). Some highly polymorphic VNTR loci have different powers of discrimination among strains from different regions; for example the discriminatory power of ETRE among strains from Jiangsu [22] and MIRU26 among strains from Shanghai [23] is much greater than among strains from Beijing. We compared the discrimination power of VNTR loci in this study with those in a previous study on Beijing strains published four years ago [24]. Jiao et al. typed 72 Beijing family strains isolated in Beijing city from 2002 to 2005 using 24 VNTR loci. It is interesting that the discrimination power and alleles of some loci were different from our results. The differences are likely due to the different populations in the distinct geographic areas where the *M. tuberculosis* strains were isolated or to differences in sampling times.

This study suffers from one main limitation. Our study based on a comparison of strains from Beijing, Shanghai and Jiangsu provinces suggests that VNTR results for *M. tuberculosis* strains differ according to geographic region. However, more data from other provinces is needed to precisely identify the nature of differences between different regions of China.

## Conclusions

Beijing family strains are the most prevalent genotypes in Beijing city. The predominance of Beijing family strains has not altered for almost twenty years. Many of the differences in allelic diversity between VNTR locus studies from different areas are likely due to the population differences in the regions from which the *M. tuberculosis* strains were isolated or to different sampling times.

## Competing interests

The authors declare that they have no competing interests.

## Authors’ contributions

BL participated in data analysis and drafted the manuscript; PZ and XZ participated in sample collection; BL and QY carried out the molecular genetic studies; HD and KW participated in the design of the study. All authors read and approved the final manuscript.

## Pre-publication history

The pre-publication history for this paper can be accessed here:

http://www.biomedcentral.com/1471-2334/12/372/prepub

## Supplementary Material

Additional file 1: Table S1 Results for the 22 VNTR loci, spoligotyping, LSP and NTF analyses of the 260 strains isolated in Beijing, China in 2009. Click here for file
